# Selection of an impedance- or magnetic field-based electro-anatomical mapping platform does not affect outcomes of outflow tract premature ventricular complex manual ablation

**DOI:** 10.1007/s00380-022-02081-4

**Published:** 2022-05-12

**Authors:** Pál Ábrahám, Mercédesz Ambrus, Szilvia Herczeg, Nándor Szegedi, Klaudia Vivien Nagy, Zoltán Salló, Péter Perge, István Osztheimer, Gábor Széplaki, Tamás Tahin, Béla Merkely, László Gellér

**Affiliations:** 1 Semmelweis University Heart and Vascular Centre, Városmajor u. 68, Budapest, 1122 Hungary; 2 Heart and Vascular Centre, Mater Private Hospital, Eccles St, Dublin 7, D07 WKW8 Ireland; 3 Royal College of Surgeons in Ireland, 23 St Stephen’s Green, Dublin 2, D02 YN77 Ireland

**Keywords:** Electro-anatomical mapping, Premature ventricular complexes, Catheter ablation, Outflow tract, Outcome

## Abstract

Comparative data are virtually missing about the performance of different electro-anatomical mapping (EAM) system platforms on outflow tract (OT) premature ventricular complex (PVC) ablation outcomes with manual ablation catheters. We aimed to compare the acute success-, complication-, and long-term recurrence rates of impedance-based (IMP) and magnetic field-based (MAG) EAM platforms in manual OT PVC ablation. Single-centre, propensity score matched data of 39–39 patients ablated for OT PVCs in 2015–17 with IMP or MAG platforms were analysed. Acute success rate, peri-procedural complications, post-ablation daily PVC burden, and long-term recurrence rates were compared on intention-to-treat basis. Acute success rate was similar in the IMP and MAG group (77 vs. 82%, *p* = 0.78). There was a single case of femoral pseudo-aneurysm and no cardiac tamponade occurred. PVC burden fell significantly from baseline 24.0% [15.0–30.0%] to 3.3% [0.25–10.5%] (*p* < 0.001) post-ablation, with no difference between EAM platforms (IMP: 2.6% [0.5–12.0%] vs. MAG: 4.0% [2.0–6.5%]; *p* = 0.60). There was no significant difference in recurrence-free survival of the intention-to-treat cohort of the IMP and MAG groups (54 vs. 60%, *p* = 0.82, respectively) during 12 months of follow-up. Ablation with the aid of both impedance- and magnetic field-based EAM platforms can considerably reduce OT PVC burden and give similar acute- and long-term freedom from arrhythmia.

## Introduction

The right- (RVOT) and left ventricular outflow tracts (LVOT) are the regions where the majority of idiopathic ventricular arrhythmias arise with premature ventricular complexes (PVC) being the most frequent of them. Other sources of outflow tract (OT) PVCs include the sinuses of the aortic valve, the pulmonary trunk, and the epicardial aspect of the OT area. While OT PVCs are overwhelmingly benign in prognosis, they can cause palpitations, pounding, and a feeling of skipped beats that could lead to significant discomfort and reduced quality of life. Moreover, Penela et al. [1] reported that a high daily PVC burden can decrease left ventricular ejection fraction (LVEF) leading to PVC-induced cardiomyopathy. Zhong et al. [2] demonstrated in their retrospective study that PVC ablation significantly reduced arrhythmia burden and improved LVEF compared with antiarrhythmic drug therapy only. Radiofrequency catheter ablation has become a feasible and efficient treatment recommended by the 2015 ESC Guidelines of ventricular arrhythmia management [3]. Long-term success of ablation not only can reverse the decline in LVEF but can also improve clinical prognosis of PVC-induced cardiomyopathy by reducing the composite of mortality, indication for heart transplant, and hospitalisation as it was proven in the landmark prospective multicentre trial by Berruezzo et al. [4].

The acute procedural success rate of OT PVC ablation is around 80–90% but long-term recurrence rates are variable in the literature, even reaching 52% in RVOT cases with a mean time to recurrence of 6.3 years according to Ventura et al. [5].

Unlike in atrial fibrillation ablation [6], the use of manual contact force sensing (CFS) catheters in OT PVC ablation does not seem to outperform traditional manual ablation catheters in reducing PVC recurrence as it has been independently pointed out by a recent retrospective multicentre study of Reichlin et al. [7] and by Ábrahám et al. [8] in a propensity-matched retrospective cohort. An explanation could be that ablation in the OT region is rather focal and activation-based in an anatomically quite variable region and it is not a matter of creating a homogenous, anatomically driven lesion set, such as in atrial fibrillation ablation. Therefore, the performance of the electro-anatomical mapping (EAM) system could theoretically influence ablation success, as well. The most widespread 3-dimensional EAM platforms are magnetic field-based (MAG) and impedance-based (IMP) in their fundamental design. There is virtually no data available on the performance and clinical outcome of different EAM systems in manual OT PVC ablation. Outcomes with the two EAM systems were only compared in remote magnetic navigation (RMN) environment by Dang et al. [9], notably with neutral results.

We aimed to compare the change in PVC burden, acute success rates, and 12 month follow-up results of MAG- and IMP-guided manual endocardial ablation on an intention-to-treat basis in a balanced patient population treated exclusively for OT PVCs.

## Methods

### Patients

We analysed observational data of a single-centre, retrospective cohort of 177 consecutive adult patients who underwent their first OT PVC ablation between 2015 and 2017 in our centre. Patients with symptoms characteristic for PVC with a high (> 15%) daily arrhythmia burden refractory to medical therapy or patients refusing to take antiarrhythmic drugs were included. Data of 177 patients underwent propensity score matching to create balanced cohorts. Informed consent was obtained from patients prior to inclusion to the study. Antiarrhythmic medication was discontinued 48 h before ablation. For lateralisation of the source of origin the V2S/V3R amplitude ratio of the OT PVC beats measured on the 12-lead ECG was used as described by Yoshida et al. [10]. The study protocol was reviewed and approved by the Institutional Review Board and was in accordance with the Declarations of Helsinki.

### Procedure and follow-up

Procedures were performed under local anaesthesia using a femoral venous and/or arterial vascular access. There was neither a transseptal approach for left-sided ablation nor a direct subxyphoid access for epicardial ablation. Three-dimensional EAM was performed in all patients to map the earliest endocardial activation of OT PVC and to guide ablation using either a MAG system: CARTO 3^®^ (Biosense Webster Inc., Diamond Bar, CA, USA) or an IMP platform: EnSite NavX^®^ (Abbott Inc., Abbott Park, IL, USA). Although the latter platform has been upgraded to a hybrid IMP + MAG system, called EnSite Precision^®^ since 2016 only cases without magnetic sensor-enabled ablation catheters were selected from 2016 to 17 for data consistency purposes of the IMP group. Only contact mapping was used.

Activation mapping identified sites for ablation with earliest local activation advancing the onset of PVC QRS by at least 30 ms. During traditional pace-mapping, a site with a minimum match of 11/12 was considered a target to energy delivery. Automated morphology matching programs, PASO^®^ (Biosense Webster Inc., Diamond Bar, CA, USA) and ScoreMap^®^ (Abbott Inc., Abbott Park, IL, USA) were also used when they became available on the relevant platform. When spontaneous, clinically relevant PVCs were absent during the procedure isoproterenol was used in 10 µg boluses for induction of the arrhythmia. Commercially available 3.5 mm open-irrigated tip radiofrequency catheters were used for mapping and ablation with or without CFS capabilities. Radiofrequency energy was delivered in 60 s duration impulses with an energy setting of 30–40 W, in power-controlled mode with a temperature limit of 43 °C. The definition of acute procedural success was complete abolition of the clinical PVC during the post-ablation waiting period after isoproterenol challenge. Arrhythmia recurrence during follow-up was defined as a > 5% daily PVC burden on a 24 h Holter-ECG. Visits and Holter-ECGs were performed at 3 months post-ablation, and then every 6 months on, or in between these periods when symptoms recurred. Left-sided ablations included PVCs originating from the LVOT region or from the left- and right sinuses of Valsalva.

### Statistical analysis

Variables included in the logistic regression model for propensity score matching were age, sex, hypertension, use of CFS catheters, and left-sided ablations. A 1:1 ratio and nearest neighbour method was used by the algorithm to create a propensity-matched cohort. A value of 0.1 was set for maximum allowed difference in matching. Continuous variables were expressed as median and interquartile ranges [25–75% IQR], whereas categorical ones in numbers and %. We compared dichotomous variables with Fisher’s exact test. Continuous variables with normal distribution were compared using t test, or by Mann–Whitney U test for the variables with non-normal distribution. We assessed normality of distribution with a Shapiro–Wilk test. Recurrence-free survival was computed by the Kaplan–Meier method and differences in recurrence rates were compared with log-rank test. We evaluated recurrence-free survival rates on an intention-to-treat basis between the MAG and IMP group, hence not only acutely successful cases were included in the analysis. We accepted a two-tailed p value of < 0.05 as statistically significant. We used SPSS Statistics 27 (IBM Corp., Armonk, NY, USA) software for calculations.

## Results

### Baseline characteristics

Out of the 177 OT PVC patients (41 IMP and 136 MAG cases) propensity matching algorithm created a group of 78 paired cases and in 99 cases matching was ineffective. 39 patients underwent IMP-guided manual ablation and 39 matched patients had a procedure with MAG guidance. The IMP and MAG groups created by matching were balanced and there was no significant difference in baseline clinical variables (Table 1). The median age of patients was 53 [42–66] years, 45 (58%) of them being female. Median LVEF was 55% [45–60%], and 36% of the cohort had a reduced (< 50%) LVEF, with no significant difference in their distribution between the two EAM groups (*p* = 0.06). The site of origin was equally represented with 39–39 (50–50%) cases localised to the RVOT and LVOT. There was no significant difference in baseline antiarrhythmic medication with beta-blockers being the most frequent in 43 (55%) patients (Table 2). All procedural parameters showed insignificant differences (Table 3). Median procedure time was 65 [55–80] min. A contralateral ablation approach, when the successful ablation site of the clinical PVC was finally in the opposite OT to the initial approach indicated by surface ECG, was needed in 27% of the cases, with no significant difference between groups (*p* = 0.31). We used pace-mapping in 58 cases (74%) and their distribution between the MAG and IMP groups did not show significant difference (*p* = 0.19) as shown in Table 3. There was no difference in median baseline PVC burden between groups whether pace-mapping was performed or not (25 vs. 26%, *p* = 0.81).

**Table 1:** Baseline clinical characteristics

	All patients (*n* = 78)	IMP (*n* = 39)	MAG (*n* = 39)	*p*
Age (year)	53 (42–66)	52 (41–65)	54 (43–67)	0.71
Female	45 (58%)	22 (56%)	23 (59%)	1.00
Diabetes	11 (14%)	5 (13%)	6 (15%)	1.00
Hypertension	42 (54%)	19 (49%)	23 (59%)	0.49
Ischaemic heart disease	13 (17%)	3 (8%)	10 (26%)	0.06
eGFR (ml/min)	77 (63–90)	81 (53–90)	76 (69–86)	0.55
Daily PVC burden (%)	24 (15–30)	20 (15–30)	25 (15–30)	0.60
LVEF (%)	55 (45–60)	47 (37–55)	58 (53–60)	0.07
PVC QRS width (ms)	160 (145–175)	160 (142–172)	160 (148–180)	0.87
Antiarrhythmics use^a^	59 (76%)	33 (85%)	25 (64%)	0.07
LVOT origin^b^	39 (50%)	19 (49%)	20 (51%)	1.00

Continuous variables are expressed as median and interquartile range

*IMP* impedance-based navigation platform, *MAG* magnetic field-based navigation platform, *eGFR* estimated glomerular filtration rate, *PVC* premature ventricular complex, *LVEF* left ventricular ejection fraction, *LVOT* left ventricular outflow tract

^a^Including beta-blockers

^b^Including all left-sided sites of origin

**Table 2:** Baseline medications

	All patients (*n* = 78)	IMP (*n* = 39)	MAG (*n* = 39)	*p*
Beta-blockers	43 (55%)	22 (56%)	21 (54%)	1.00
Verapamil	5 (6%)	4 (10%)	1 (3%)	0.36
Amiodarone	4 (5%)	2 (5%)	2 (5%)	1.00
Sotalol	1 (1%)	0 (0%)	1 (3%)	1.00
Propafenon	2 (3%)	1 (3%)	1 (3%)	1.00
ACEI/ARB	35 (45%)	18 (46%)	17 (44%)	1.00
MRA	11 (14%)	5 (13%)	6 (15%)	1.00
VKA	7 (9%)	3 (7%)	4 (10%)	0.62
NOAC	1 (1%)	0 (0%)	1 (3%)	1.00
ASA	19 (24%)	8 (21%)	11 (28%)	0.59
Clopidogrel	3 (4%)	1 (3%)	2 (5%)	1.00

*IMP* impedance-based navigation platform, *MAG* magnetic field-based navigation platform, *ACEI* angiotensin converting enzyme, *ARB* angiotensin receptor inhibitor, *ASA* acetyl-salicylic acid, *MRA* mineralocorticoid receptor antagonist, *NOAC* non vitamin-K oral anticoagulant, *VKA* vitamin-K antagonist

**Table 3:** Procedural parameters and complications

	All patients (*n* = 78)	IMP (*n* = 39)	MAG (*n* = 39)	*p*
Procedural parameters				
Procedure time (min)	65 (55–80)	65 (55–50)	61 (51–88)	0.70
Fluoro-time (min)	2.5 (1.6–4.1)	2.1 (1.7–3.5)	3.2 (1.7–4.6)	0.06
RF time (s)	339 (185–651)	386 (185–661)	288 (181–611)	0.90
RF applications (N)	6 (3–11)	9 (3–11)	5 (4–12)	0.80
Bilateral ablation	21 (27%)	8 (21%)	13 (33%)	0.31
RF above leaflet^a^	12 (15%)	8 (21%)	4 (10%)	0.35
CFS	8 (10%)	4 (10%)	4 (10%)	1.00
Pace-mapping used	58 (74%)	26 (67%)	32 (82%)	0.19
Acute success rate	62 (79%)	30 (77%)	32 (82%)	0.78
Complications				
Groin haematoma	2 (3%)	2 (5%)	0 (0%)	0.49
Pseudo-aneurysm	1 (1%)	1 (3%)	0 (0%)	1.00
Cardiac tamponade	0 (0%)	0 (0%)	0 (0%)	1.00
Pericardial effusion	1 (1%)	0 (0%)	1 (3%)	1.00
Stroke/TIA	0 (0%)	0 (0%)	0 (0%)	1.00

Continuous variables are expressed as median and interquartile range

*IMP* impedance-based navigation platform, *MAG* magnetic field-based navigation platform, *CFS* contact force sensing ablation catheter, *RF* radiofrequency, *TIA* transitory ischemic attack

^a^Ablation target above the valvular planes

### Acute success

Acute success rates were not statistically different between the MAG and IMP groups with 32 (82%) and 30 (77%) cases (*p* = 0.78), respectively (Table 3).

### Clinical follow-up

**Fig. 1:**
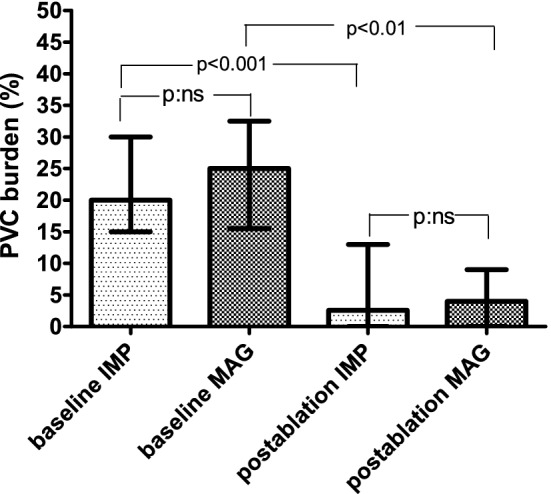
Comparison of daily baseline and post-ablation PVC burden grouped by EAM platforms. Graph displays medians and interquartile ranges. *EAM* electro-anatomical mapping, *IMP* impedance-based navigation platform *MAG* magnetic field-based navigation platform, *PVC* premature ventricular complex

During the follow-up time of 12 months, 4 patients (5%) were lost to follow-up. These patients were reported being alive on telephone interview, but declined to take part in follow-up visits, therefore, they were excluded from the analysis of recurrence. The 24 h PVC burden plunged significantly from a baseline median value of 24.0% [15.0–30.0%] to 3.3% [0.25–10.5%], *p* < 0.001. The magnitude of reduction was similar in the MAG (from 25.0% [15.0–30.0%] to 4.0% [2.0–6.5%]; *p* < 0.01) and in the IMP group (from 20.0% [15.0–30.0%] to 2.6% [0.5–12.0%]; *p* < 0.001) without a significant between-group difference (*p* = 0.60), as one can see on Fig. 1.

**Fig. 2:**
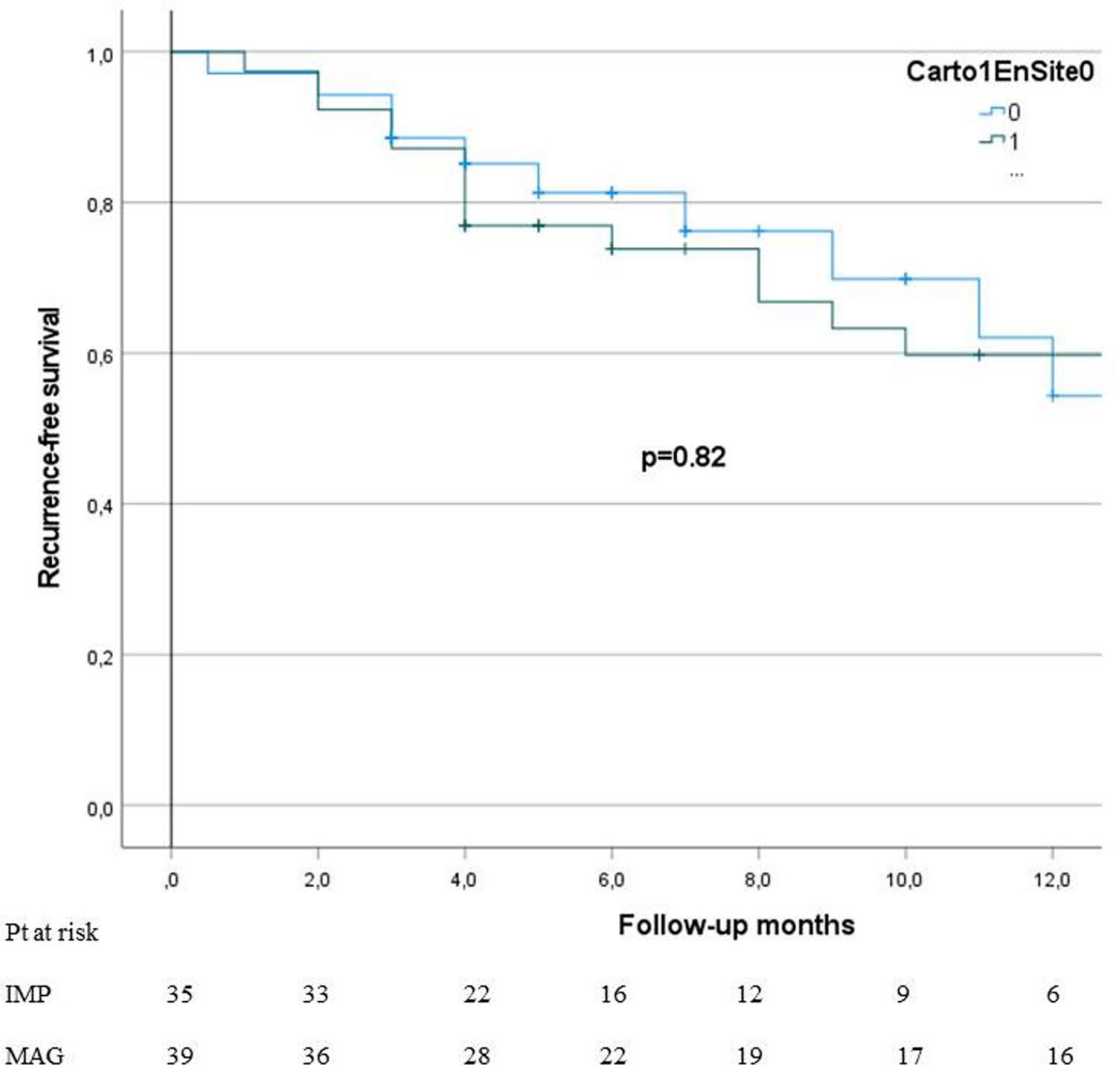
Intention-to-treat-based Kaplan–Meier curves of PVC recurrence-free survival stratified by the EAM platforms used. Carto® stands for MAG and EnSite^®^ stands for IMP platform. *P* represents log-rank test for comparison

Including all but lost patients into the analysis on an intention-to-treat basis there was no significant difference in recurrence-free survival rates between the MAG (60%) and IMP (54%) groups (log-rank *p* = 0.82) over 12 months of follow-up, as depicted on Fig. 2.

### Complications

Access-site complications included two (2.6%) groin haematomas and one (1.3%) pseudo-aneurysm, the latter requiring surgical closure and transfusion (Table 3). No patient developed cardiac tamponade just a hemodynamically insignificant pericardial effusion occurred in a single case post-ablation. There was no procedure-related death.

## Discussion

Precise electro-anatomical activation mapping is fundamental in targeting foci with earliest activation and in achieving a durable success after OT PVC ablation. Spatial resolution and accuracy of mapping has already been validated both in MAG- and IMP-based platforms by using a phantom model by Bourier et al. [11]. They found that the 3D localisation offset of mapped points against the phantom-based reality with CT-scan segmentation was 1.62 ± 0.77 mm in CARTO3^®^ and 2.02 ± 1.21 mm in EnSite^®^, the difference being clinically irrelevant.

The performance of MAG and IMP platforms in OT PVC ablation was only tested in RMN environment, but not with traditional manual ablation catheters. Dang et al. [9] pointed it out in their 43 patients that there was no difference in procedural parameters including ablation duration and X-ray exposure between EnSite Precision^®^ and CARTO3^®^ systems used with magnetic navigation. Long-term success rates were also comparable (86.4% vs. 81.0%, *p* = 0.631) during their 16.2 ± 6.2 months of follow-up. Our novel results in OT PVC ablation with manual catheters are in accordance with their findings. Procedural parameters, the magnitude of decline in PVC burden, and 12 month follow-up results did not show any significant difference between the MAG and IMP groups in our propensity-matched cohort of 78 patients. It is worth mentioning that we had a balanced proportion of left- and right sided ablation targets and the use of CFS catheters was also represented proportionally in both groups.

Mapping in the OT region could be quite a challenge, therefore, more and more sophisticated features were developed and integrated into 3D EAM systems in the last decade.

High-density mapping features like Confidense^®^ in MAG (launched in June 2015) and EnSite Precision^®^ in IMP system (launched in January 2016) create much more detailed anatomical voltage- and activation maps with a high level of automation. In the case of rarely occurring PVCs, pace-mapping can help target the focus albeit with less spatial accuracy than activation mapping [12]. Development of the MAG platform in pace-mapping resulted in the PASO module to better quantify surface ECG QRS morphology match in 2013. ScoreMap has been serving the same purpose in the IMP platform since 2016. Year 2015 was dominated in PVC ablation in our centre by the MAG platform in this propensity-matched cohort with 84%, and our practice remarkably shifted towards the IMP platform from 2016. As much as 74% and 88% of the cases in 2016 and 2017 were performed by IMP-based mapping.

### Complications

Procedure-related complications were infrequent as they occurred in four cases (5%) in our cohort and only one patient (1.3%) had a major complication that required surgical intervention at the femoral access site and transfusion. Complication rates were not statistically different regarding EAM platforms, as shown in Table 3. De Vires et al. [13] reported a 10% overall and 3% major complication rate in their 73 patients treated with catheter ablation for outflow tract arrhythmias.

### Limitations

A major limitation of this work lies in its non-randomized nature that might give chance to an unmeasured confounder. Nevertheless, the balanced proportion of 11 baseline clinical variables and 8 procedural parameters, and the similar pattern of antiarrhythmic drug use probably could reduce this chance to a minimum. It is important to emphasize that our sample size was quite small to arrive to any solid conclusion regarding complications when comparing manual MAG and IMP-guided ablation in the OT region.

Since the introduction of EnSite Precision^®^ a hybrid 3D EAM system has been operating in this platform, where the incorporation of a magnetic sensor further enhanced mapping precision. Therefore, one cannot label this novel hybrid mapping platform as purely IMP based. On the other hand, we deliberately selected cases without sensor-enabled ablation catheters to the IMP group to keep this group purely IMP-based in mapping. As a consequence, the full functionality of the hybrid Precision^®^ module cannot be assessed in this work.

By the time those ablations were performed there had not been other EAM platforms available than CARTO 3^®^ (Biosense Webster Inc., Diamond Bar, CA, USA) and EnSite^®^ (Abbott Inc. Abbott Park, IL, USA) in our institution. Because RHYTHMIA^®^ (Boston Scientific Inc., Marlborough, MA, USA) was installed later in our lab, our comparative work could not cover the full spectrum of commercially available high-resolution mapping solutions.

We did not use non-contact mapping at all, therefore, our ablation results with sparsely occurring PVCs might have been affected by this fact.

## Conclusions

To the best of our knowledge, this is the first work that directly compares clinical outcomes of the most widespread MAG and IMP EAM platform-based catheter ablation techniques in OT PVC ablation. In our propensity-matched population of 78 patients, there was no difference in OT PVC ablation outcomes regardless of the mapping platform used. What makes the value of our work is the balanced inclusion of patients with homogenous baseline characteristics between the MAG and IMP group. Most noteworthy, the representation of right- and left-sided cases was also equal.
